# Multiple genetic variants involved in both autoimmunity and autoinflammation detected in Chinese patients with sporadic Meniere's disease: a preliminary study

**DOI:** 10.3389/fneur.2023.1159658

**Published:** 2023-05-18

**Authors:** Jing Zou, Guoping Zhang, Hongbin Li, Zikai Zhao, Qing Zhang, Ilmari Pyykkö, Antti Mäkitie

**Affiliations:** ^1^Department of Otolaryngology-Head and Neck Surgery, Changhai Hospital, Second Military Medical University, Shanghai, China; ^2^Research Program in Systems Oncology, Department of Otorhinolaryngology-Head and Neck Surgery, Helsinki University Hospital and University of Helsinki, Helsinki, Finland; ^3^Hearing and Balance Research Unit, Field of Otolaryngology, School of Medicine, Faculty of Medicine and Health Technology, Tampere University, Tampere, Finland

**Keywords:** Meniere's disease, immunology, genetics, mutation, diagnostics

## Abstract

**Background:**

The mechanisms of Meniere's disease (MD) remain largely unknown. The purpose of this study was to identify possible genetic variants associated with immune regulation in MD.

**Methods:**

The whole immune genome of 16 Chinese patients diagnosed with sporadic MD was sequenced using next-generation sequencing.

**Results:**

Definite pathological variants of *MEFV* (c.1223G>A, c.1105C>T), *COL7A1* (c.5287C>T), and *ADA* (c.445C>T) contributing to the clinical phenotype were found in three patients. Limited and likely pathological variants of *TLR3* (c.2228G>A) and *RAB27A* (c.560G>A) were detected in one patient each. The following definite pathological variants impairing the structure and function of translated proteins were detected in 10 patients, and multigene variants occurred in five patients: *PRF1* (*c.710C*>*A*), *UNC13D* (c.1228A>C), *COLEC11* (c.169C>T), *RAG2* (c.200G>C), *BLM* (c.1937G>T), *RNF31* (c.2533G>A), *FAT4* (c.11498A>G), *PEPD* (c.788A>G), *TNFSF12* (c.470G>A), *VPS13B* (c.11972A>T), *TNFRSF13B* (c.226G>A), *ERCC6L2* (c.4613A>G), *TLR3* (c.2228G>A), *ADA* (c.445C>T), *PEPD* (c.151G>A), and *MOGS* (c.2470G>A). The following limited pathological variants impairing the structure and function of translated proteins were detected in five patients, with double gene variants identified in one patient: *EXTL3* (c.1396G>A), *MTHFD1* (c.2057G>A), *FANCA* (c.2039T>C), *LPIN2* (c.1814C>T), *NBAS* (c.4049T>C), and *FCN3* (c.734G>A).

**Conclusion:**

Patients with sporadic MD carry multiple genetic variants involved in multiple steps of immune regulation, which might render patients susceptible to developing inflammation via both autoimmune and autoinflammation mechanisms upon internal stress.

## 1. Introduction

The mechanisms of Meniere's disease (MD), which is characterized by episodic vertigo, fluctuant hearing loss, tinnitus, and aural fullness, remain largely unknown. Allergy was reported to be associated with MD as early as the 1940's (1, 2), and the autoimmune mechanism was later indicated (3, 4). The excellent therapeutic effect of intratympanic corticosteroids further supports an immune-inflammatory reaction associated with pathological progression (5). Although autoimmunity has been identified in both unilateral and bilateral MD subgroups, the possible immune-inflammatory mechanism has not been evaluated in subgroups according to the classification method (6, 7). High basal levels of IL-1β were detected in the supernatant of 24 out of 113 MD patients (21%), and 20 out of the 24 patients were diagnosed with sporadic MD. Among the 24 patients with high basal levels of IL-1β, 21 (87.5%) showed elevated levels of IL-6, 19 (79.2%) had higher levels of TNF-α, and 17 (70.8%) demonstrated an elevation of IL-1R antagonist levels (8). There is a possibility that the autoinflammatory mechanism was involved in those MD patients with high levels of IL-1β. In contrast to autoimmune diseases that are characterized by the production of specific autoantibodies and the activation of T cells and B cells, autoinflammatory diseases are at the other end and mediated by inflammasomes that further introduce the secretion of active IL-1β (9).

Variants in inflammation-related genes were detected in sporadic MD patients (10). Several clinical subgroups of MD have been reported, but the criteria for genetic variants have not been defined (6, 7). MD patients with a familial history and autoimmunity may have a unique genetic background, and the criteria for MD with autoinflammation have not been defined (10). Zou reported a case of refractory MD demonstrating autoinflammatory characteristics supported by both genotype and associated phenotype and the effects of delivering high-dose steroids to the surface of the intact endolymphatic sac and incus (11). McDermott and Kastner first introduced the term “autoinflammation” in 1999, describing the critical role of impaired membrane TNFR1 clearance and diminished shedding of the potentially antagonistic soluble receptor in autosomal dominant periodic fever syndromes characterized by unexplained fever episodes and severe localized inflammation (12). Six categories of autoinflammatory diseases have been defined based on molecular pathophysiology. However, a mixture of autoinflammation and autoimmunity might be developed during the late stage of diseases possibly mediated through classical dendritic and plasmacytoid dendritic cells-induced differentiation of Th1 and Th17 effector T cell subsets (13). A thorough investigation into the autoinflammatory mechanisms of MD remains warranted.

We aimed to identify possible genetic variants associated with immune regulation, including autoinflammatory and autoimmune mechanisms involved in the occurrence of MD. The whole immune genome of Chinese patients with sporadic MD was, thus, sequenced in the present study.

## 2. Materials and methods

### 2.1. Patient cohort

The protocol was reviewed and approved by the ethics committee of Shanghai Changhai Hospital (CHEC2020-107). Patients who visited the author, JZ, at the Department of Otolaryngology—Head and Neck Surgery, Changhai Hospital, Second Military Medical University, from July 2018 to June 2022, were screened. The inclusion criteria were as follows: (1) diagnosis of definite MD made according to the 2015 criteria of the Barany Society (14) and (2) endolymphatic hydrops (EH) was confirmed using gadolinium enhancement MRI. The exclusion criteria were as follows: vertigo of other origins, such as benign paroxysmal positional vertigo, and diseases of the central nervous system. Since familial MD is a heterogeneous condition, some individuals presenting with a partial syndrome (i.e., episodic vertigo without low-frequency sensorineural hearing loss) fail to meet the diagnostic criteria of MD (15). Familial history ruled out familial clustering of the patients in the current study. Subgroups of unilateral and bilateral MD patients were defined according to previous reports (6, 7). In total, 16 patients diagnosed with definite MD were enrolled (Table 1). The data of two previously reported patients were also included in the current study in order to draw conclusions from accumulated information (11, 16).

**Table 1:** EH detected in MD patients using MRI 24 h after intratympanic injection of Gd-DTPA.

**ID**	**CoEH-L**	**CoEH-R**	**VeEH-L**	**VeEH-R**	**MRI protocol**	**Side**	Subgroup^*^
*A*	0	2	0	3	3D real IR	R	Type 1
*B*	1	2	0	3	hT_2_W-FLAIR	B	Type 1
*C*	0	0	0	2	hT2W-FLAIR	R	Type 2
*D*	2	0	3	0	hT_2_W-FLAIR	B	Type 2
*E*	0	2	1	3	MIIRMR	R	Type 1
*F*	1	2	0	3	MIIRMR	R	Type 1
*G*	No uptake	2	3	Leakage	hT_2_W-FLAIR	B	Type 1
*H*	1	2	2	0	MIIRMR	B	Type 1
*I*	No uptake	2	No uptake	3	MIIRMR+hT2W-FLAIR	B	Type 1
*J*	1	0	2	0	MIIRMR+hT2W-FLAIR	L	Type 1
*K*	1	2	2	3	MIIRMR	B	Type 1
*L*	0	2	0	1	hT_2_W-FLAIR-ZFI	R	Type 1
*M*	2	2	3	3	hT_2_W-FLAIR-ZFI	B	Type 1
*N*	2	2	1	0	hT_2_W-FLAIR-ZFI	B	Type 2
*O*	0	2	0	3	hT_2_W-FLAIR-ZFI	R	Type 1
*P*	0	2	0	2	hT_2_W-FLAIR-ZFI	R	Type 1

* Subgroups of unilateral and bilateral MD patients were defined according to previous reports (2, 3).

3D real IR, Three-dimensional fluid-attenuated inversion recovery with real reconstruction; CoEH-L, CoEH-R, cochlear endolymphatic hydrops on the left, and right side; hT_2_W-FLAIR, heavily T_2_-weighted three-dimensional fluid-attenuated inversion recovery with magnitude reconstruction; hT_2_W-FLAIR-ZFI, hT_2_W-FLAIR reconstructed with magnitude and zero-filled interpolation; MIIRMR, medium inversion time inversion recovery imaging with magnitude reconstruction; VeEH-L, VeEH-R, vestibular endolymphatic hydrops on the left and right side.

Patients were treated mainly with intratympanic dexamethasone (ITDex) and additional high-dose steroid delivery to the surface of the intact endolymphatic sac and incus (ESCS) when they were unsatisfied with ITDex (Table 1). Patient E was treated with endolymphatic sac decompression by another doctor without effect, received ITDex and ESCS without effect, and was finally treated with the triple semicircular canal plugging by another doctor. Patient L was treated by endolymphatic sac shunt surgery in another hospital due to unsatisfactory ITDex and suffered from severe tinnitus after surgery. The following criteria were used for evaluating the outcome of therapy: V2 = no vertigo attack and normal balance, V1 = reduced vertigo, V0 = vertigo remained the same, V(-1) = vertigo became worse; H2 = complete recovery in hearing, H1 = hearing improved >10 dB (mean pure tone at 0.5, 1.0, and 2.0 kHz), H0 = hearing remained the same, and H(-1) = hearing became worse with a mean value >10 dB; T2 = tinnitus disappeared, T1 = reduced tinnitus, T0 = tinnitus remained the same, and T(-1) = tinnitus became worse; F2 = fullness disappeared, F1 = reduced fullness, F0 = fullness remained the same, and F(-1) = fullness became worse.

### 2.2. MRI

To detect EH in MD patients, MRI was performed 24 h after intratympanic injection of Gd-DTPA at a previously reported dose using a 3T MR system (MAGNETOM Skyra, Siemens Healthcare, Erlangen, Germany) equipped with a 20-channel Tim 4G head/neck coil (17). Either hT_2_W-FLAIR, MIIRMR, or hT_2_W-FLAIR-ZFI was applied for imaging as the protocols were updated three times during the research period (Table 1) (18, 19). The SPACE sequence was also applied to image potential inner ear fibrosis or vestibular schwannoma (17, 18). EH was graded according to previous reports that applied a three-stage method for cochlear hydrops and a four-stage scale for vestibular hydrops based on morphological changes (20, 21). A modification was made in the grading of 4th-grade vestibular hydrops, defining this grade when the bright peripheral rim of the surrounding perilymphatic space is largely absent instead of when the vestibular perilymphatic enhancement becomes invisible.

### 2.3. Genetic analysis

Genomic DNA was extracted from peripheral blood samples from all patients. Whole exomes including 269, 349, or 423 candidate genes (Table 2) potentially involved in autoinflammatory diseases, autoimmune diseases, and other diseases associated with disorders of immune regulation were selected according to Human Phenotype Ontology (http://human-phenotype-ontology.github.io/) and sequenced using target gene capture and next-generation sequencing using a previously reported protocol (22). Data were analyzed using bwa-0.7.10, samtools-1.0, picard-tools-1.119, bamtools-2.3.0, Genome Analysis TK-3.3.0, and Annovar-2014-11-12. REVEL (Rare Exome Variant Ensemble Learner) software was used to predict the pathogenicity of missense variants based on individual tools regarding impairing the structure and function of translated proteins (23). The following datasets were used as references: 1,000 genomes, NHLBI GO Exome Sequencing Project (ESP6500), the Exome Aggregation Consortium (ExAC), the Exome Aggregation Consortium-East Asian (ExAC-EAS), and the in-house Chinese reference population data of MyGenostics Inc. (Beijing, China) that was archived from 2,273 normal individuals. The clinical significance of sequence variants was interpreted according to the Joint Consensus Recommendation of the American College of Medical Genetics and Genomics and the Association for Molecular Pathology published in 2015 (24). The definite pathological variants associated with clinical phenotype and the structure and function of translated proteins as well as variants with autosomal dominant inheritance were further validated using Sanger sequencing.

**Table 2:** Pathogenicity of heterozygote allelic variants in patients with MD detected using targeted next-gene sequencing.

**ID**	**Sides**	**Ver**	**REVEL-P/Freq-normal^#^/Freq-Chinese^##^**	**REVEL-LP/Freq-normal^#^/Freq-Chinese^##^**	Clinical-P^*^/Freq-normal^#^/Freq-Chinese^##^	Clinical-LP^*^/Freq-normal^#^/Freq-Chinese^##^	Clinical-LikP^*^/Freq-normal^#^/Freq-Chinese^##^
*A*	U	269	*PRF1* (c.710C>A/UnK/-)^&^, UNC13D (c.1228A>C/0.01440/0.0154)^&^				
*B*	B	269	*COLEC11* (c.169C>T/0.00810/0.00528)^$^				
*C*	U	269	*RAG2* (c.200G>C/UnK/-)^$^				
*D*	B	269	*BLM* (c.1937G>T/UnK/-)^$^, *RNF31* (c.2533G>A/0.00450/U)(c.2986G>A)^$$^				
*E*	U	349					
*F*	U	349			*MEFV* (c.1223G>A/0.05410/0.04289, c.1105C>T/0.07160/0.05873)^$^		
*G*	B	349					
*H*	B	349					
*I*	B	349	*FAT4* (c.11498A>G/0.00030/UnK), *PEPD* (c.788A>G/UnK/-), *TNFSF12* (c.470G>A/ 0.00008/-)				
*J*	U	349	*VPS13B* (c.11972A>T/0.00340/0.0066)^$^				
*K*	B	349	*TNFRSF13B* (c.226G>A/0.00240/0.00594)^$^, *ERCC6L2* (c.4613A>G/UnK/-)^$^				
*L*	U	423		*EXTL3* (c.1396G>A/0.0005/0.00044)			*RAB27A* (c.560G>A/0.0070675/0.00132)
*M*	B	423		*MTHFD1* (c.2057G>A/0.0001997/-)	*COL7A1* (c.5287C>T/0.000004/-)^$^		
*N*	B	423	*TLR3* (c.2228G>A/0.000641/-)^$^	*FANCA* (c.2039T>C/UnK/-), *LPIN2* (c.1814C>T/0.0001/0.00088)		*TLR3* (c.2228G>A/0.000641/-)	
*O*	U	423	*ADA* (c.445C>T/0.0000319/ 0.00022)^$^, *PEPD* (c.151G>A/0.0038462/0.00022)^$^	*NBAS* (c.4049T>C/0.000004/-)^$^	*ADA* (c.445C>T/0.0000319/0.00022)^$^		
*P*	U	423	*MOGS* (c.2470G>A/0.0000557/0.00022)^$^	*FCN3* (c.734G>A/0.0006/0.00066)			

# Freq-normal: frequencies of the gene variant in the normal population, the highest frequency was taken among 1,000 genomes, ESP6500 (NHLBI Exome Sequencing Project), EXAC (The Exome Aggregation Consortium), and EXAC-EAS (EXAC of 4000 eastern Asian descent).

## Freq-Chinese: frequencies of a gene variant in the in-house Chinese reference population data of MyGenostics Inc. (Beijing, China) that was archived from 2,273 normal individuals.

* Following the Joint Consensus Recommendation of the American College of Medical Genetics and Genomics and the Association for Molecular Pathology published in 2015.

& Mutations were confirmed using Sanger sequencing in both the patient and her daughter.

$ Mutations were confirmed using Sanger sequencing in the patient.

$$ Mutated sequences were different in Sanger sequencing from that obtained using next-generation sequencing.

B, bilateral MD; Clinical-LP, limited pathology regarding clinical phenotype; Clinical-LikP, likely pathology regarding clinical phenotype; Clinical-P, definite pathology regarding clinical phenotype; REVEL-LP, variants with limited pathology on the structure and function of translated proteins predicted using rare exome variant ensemble learner (REVEL) software; REVEL-P, variants with definite pathology on the structure and function of translated proteins predicted using REVEL software; U, unilateral MD; UnK, unknown; Ver, version of gene sequencing with various candidate genes; (-), no mutation was detected.

## 3. Results

### 3.1. Clinical and MRI characteristics of MD

**Figure 1:**
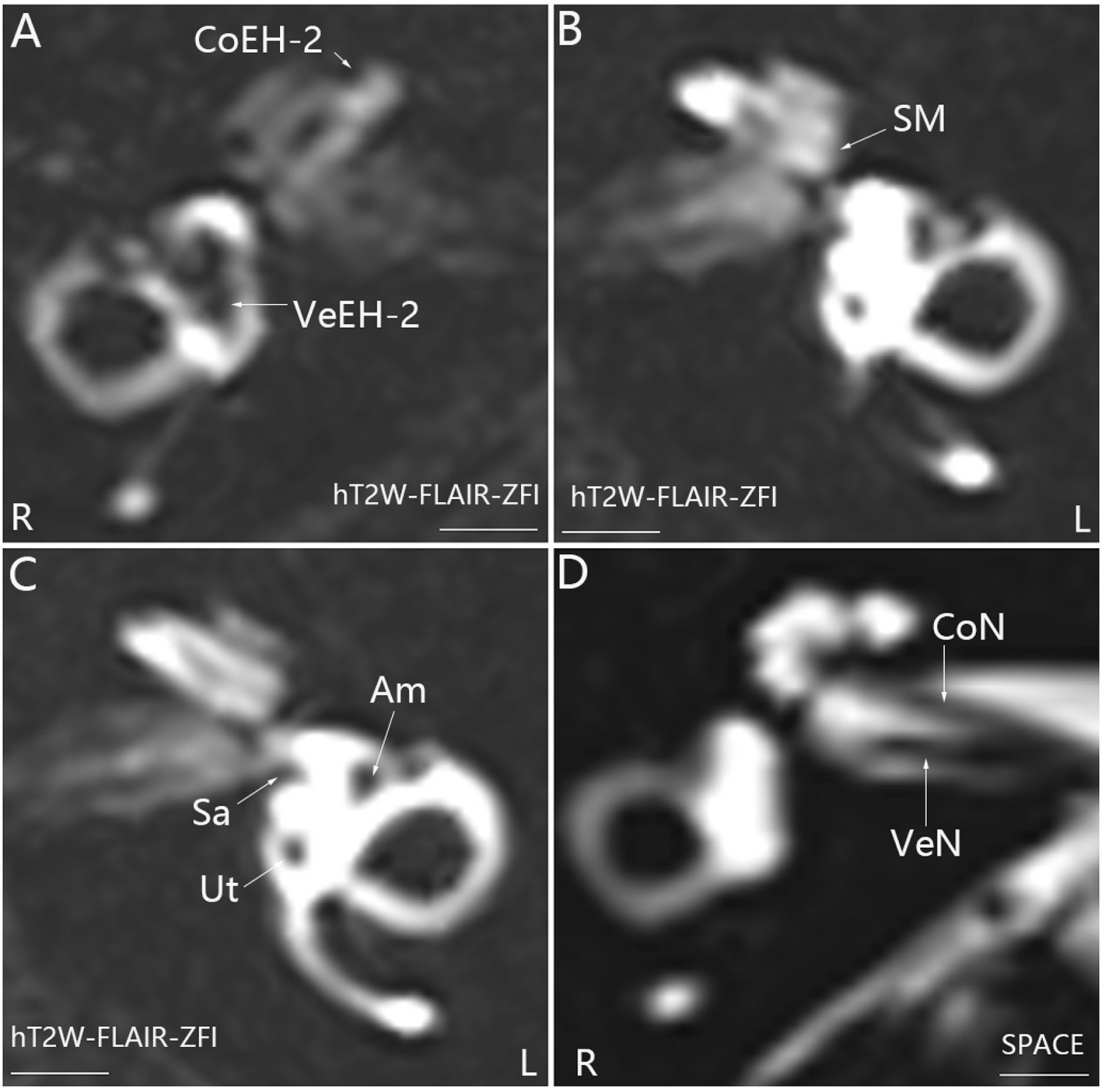
Representative MRI demonstrating EH in the right ear (R) in patient *P* with definite MD on the right side **(A)**. MR images were taken using the hT_2_W-FLAIR-ZFI sequence 24 h after intratympanic injection of 0.1 ml of 20-fold-diluted Gd-DTPA that was delivered onto the posterior upper part of the tympanic medial wall on both sides. There was no EH in the left ear (L) **(B, C)**. Neither inner ear fibrosis nor vestibular schwannoma was found on the ipsilateral side imaged using the SPACE sequence **(D)**. Am, ampulla; CoEH-2, grade 2 cochlear EH; CoN, cochlear nerve; Sa, saccule; Ut, utricle; VeEH-2, grade 2 vestibular EH; VeN, vestibular nerve. Scale bar = 3.0 mm.

**Figure 2:**
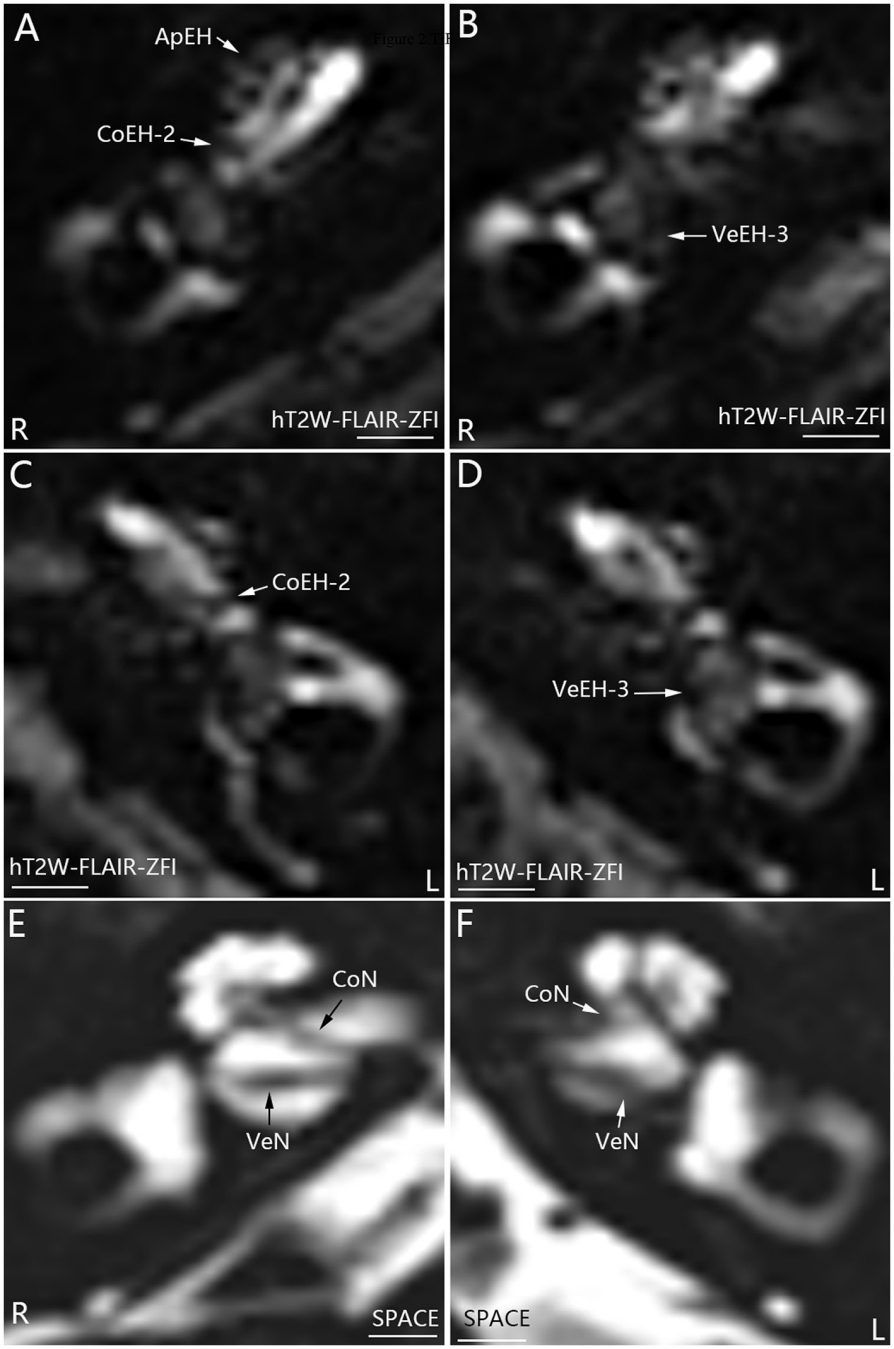
Representative MRI demonstrating EH in both ears in patient *M* with definite bilateral MD **(A–D)**. MR images were taken using the hT_2_W-FLAIR-ZFI sequence 24 h after intratympanic injection of 0.1 ml of 20-fold-diluted Gd-DTPA on both sides. Apical EH (ApEH) was also observed in the right ear (R) **(A)** but not in the left ear (L) **(C)**. Neither inner ear fibrosis nor vestibular schwannoma was observed on either side imaged using the SPACE sequence **(E, F)**. CoEH-2, grade 2 cochlear EH; CoN, cochlear nerve; VeEH-3, grade 3 vestibular EH; VeN, vestibular nerve. Scale bar = 3.0 mm.

**Figure 3:**
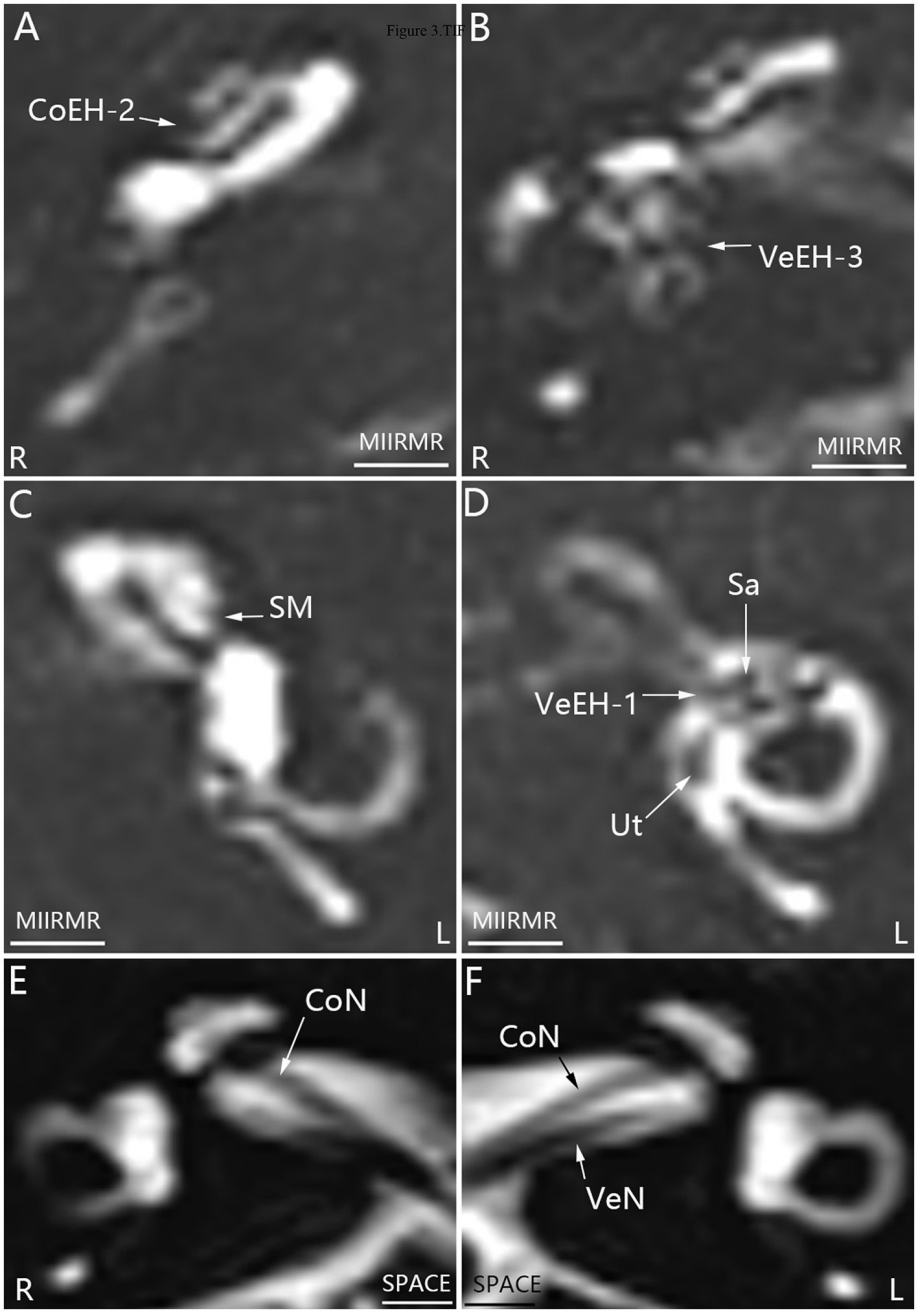
Representative MRI demonstrating EH in both ears in patient *E* with definite MD on the right side **(A, B, D)**. MR images were taken using the MIIRMR sequence 24 h after intratympanic injection of 0.1 ml of 20-fold-diluted Gd-DTPA on both sides. There was no cochlear EH in the right ear (R) **(C)**. Neither inner ear fibrosis nor vestibular schwannoma was found on the ipsilateral side imaged using the SPACE sequence **(E, F)**. CoEH-2, grade 2 cochlear EH; CoN, cochlear nerve; L, left ear; Sa, saccule; Ut, utricle; VeEH-1 and VeEH-3, grade 1 and 3 vestibular EH; VeN, vestibular nerve. Scale bar = 3.0 mm.

All 16 patients were diagnosed with definite MD; eight patients had bilateral MD, and eight patients had unilateral MD. Various grades of EH were detected in 20 cochleae and 19 vestibules in 24 affected ears, and either cochlear or vestibular EH was detected in 23 affected ears (Figures 1, 2). Mild (grade 1) EH was found in one cochlea and one vestibule each out of seven contralateral ears (Figure 3). EH was absent in one ear of a patient with bilateral MD. Enhancement was not detected in one ear of another patient with bilateral MD (Table 1). There was a significant correlation between cochlear EH and vestibular EH, between cochlear EH and side, and between vestibular EH and side (*p* < 0.01, Spearman rho).

### 3.2. Genetic variants in MD

Regarding contribution to clinical phenotype, the following definite pathological variants were found in three out of 16 patients: *MEFV* (c.1223G > A, c.1105C > T), *COL7A1* (c.5287C > T), and *ADA* (c.445C > T). The likely limited pathological variants *TLR3* (c.2228G > A) and *RAB27A* (c.560G > A) were each detected in one of the 16 patients (Table 2). The patient (M) carrying *COL7A1* variation, which causes R mutation in arginine at 1,763 which became a stop codon and terminate the following translation, had bilateral severe to profound hearing loss with the absence of distortion-product otoacoustic emissions but detectable auditory brainstem response. In electrocochleography, action and summating potentials were detected in the left ear but absent in the right ear. ocular vestibular evoked myogenic potentials (oVEMP) was normally recorded in the left ear but absent in the right ear. video head impulse test (vHIT) showed a reduced gain in vestibulo-ocular response with significant corrective saccades in three planes. Caloric testing demonstrated a reduction in the right ear.

Regarding impairments in the structure and function of translated proteins, definite pathological variants were detected in 10 out of 16 patients, and multigene variants occurred in five patients (Table 2). The variants were as follows: *PRF1* (*c.710C*>*A*), *UNC13D* (c.1228A>C), *COLEC11* (c.169C>T), *RAG2* (c.200G>C), *BLM* (c.1937G>T), *RNF31* (c.2533G>A), *FAT4* (c.11498A>G), *PEPD* (c.788A>G), *TNFSF12* (c.470G>A), *VPS13B* (c.11972A>T), *TNFRSF13B* (c.226G>A), *ERCC6L2* (c.4613A>G), *TLR3* (c.2228G>A), *ADA* (c.445C>T), *PEPD* (c.151G>A), and *MOGS* (c.2470G>A). Limited pathological variants were detected in five out of 16 patients, and double gene variants appeared in one patient. The variants were as follows: *EXTL3* (c.1396G>A), *MTHFD1* (c.2057G>A), *FANCA* (c.2039T>C), *LPIN2* (c.1814C>T), *NBAS* (c.4049T>C), and *FCN3* (c.734G>A).

Several variants listed as benign or uncertain heterozygote allelic variants in the currently available database were also detected in all 16 patients (Table 3). Variants with autosomal dominant inheritance were detected in 13 out of 16 patients, and eight patients had multigene involvement. Variants of the *MEFV* gene were found in six patients, and three patients had variants in multiple loci. Heterozygote variants with autosomal recessive inheritance were detected in 15 out of 16 patients, and 14 patients had multigene variants. A variant with X-linked recessive inheritance, *IL2RG* (c.1058A>C), was detected in one out of 16 patients. Variants with uncertain inheritance were found in three out of 16 patients, including *TIRAP* (c.671G>A), *POLE* (c.504A>T), and *TLR4* (c.910T>C).

**Table 3:** Benign/uncertain heterozygote allelic variants in patients with MD detected using targeted next-gene sequencing.

**ID**	**Sides**	**Ver**	**AD/Freq-normal^&^/Freq-Chinese^&&^**	**AR/Freq-normal^&^/Freq-Chinese^&&^**	**XLR/Freq-normal^&^/Freq-Chinese^&&^**	Uncertain^*^/Freq-normal^&^/Freq-Chinese^&&^
*A*	U	269		*C6* (c.823G>T/0.00160/0.00132), *ITCH* (c.1934A>G/0.00050/0.00022)^$^, *JAK3* (c.2678C>T/0.00570/0.00022)^$^, *SLC29A3* (c.1099G>A/0.00002/-)^$^, *SMARCAL1* (c.341G>A/0.01522/0.01518)		
*B*	B	269	*MEFV* (c.442G>C/0.31500/0.25781, c.416C>A/0.00340/0.00044)^∧^	*CD19* (c.1646G>C/UnK/-), *INO80* (c.1208G>A/UnK/-), *TRNT1* (c.67C>G/0.01940/0.01276)		
*C*	U	269	*POLE* (c.520G>A/UnK/-)^∧^	*DOCK2* (c.5335A>T/0.01270/0.01276), *IGLL1* (c.223G>A/0.00652/0.00814), *RFXAP* (c.232G>C/UnK/-), *SMARCAL1* (c.341G>A/0.01522/0.01518)		
*D*	B	269	*MEFV* (c.1223G>A/0.05410/0.04289, c.1105C>T/0.07160/0.05873)^∧^			
*E*	U	349		*IL17RC* (c.167G>A/0.01340/0.00946)		*TIRAP* (c.671G>A/0.00120/0.00286)
*F*	U	349	*NOD2* (c.1411C>T/0.01440/0.01364)^∧^, *ATM* (c.6095+8G>T/0.00030/0.00022) (splicing)	*C2* (c.103C>T/0.01110/UnK), *SPINK5* (c.1431-9T>G/UnK/-) (splicing), *VPS13B* (c.3562A>G/UnK/-)		
*G*	B	349	*RTEL1* (c.1519G>A/0.00010/-)^∧^, *COL7A1* (c.1798G>C/0.00090/0.00044)^∧^	*CARD9* (c.863G>A/0.00020/-), *CD79A* (c.301G>A/0.00170/0.0033), *CLPB* (c.619A>G/0.00850/0.0088), *EXTL3* (c.1108G>A/0.00004/-), *FAT4* (c.4403A>G/0.00050/0.00088), *FOXN1* (c.689C>G/0.00140/0.00132), *IKBKB* (c.754-7G>A/0.00120/UnK) (splicing), *SLC29A3* (c.488G>T/0.01240/0.02134), *STAT2* (c.269T>C/0.00020/0.00044), *TFRC* (c.95A>G/0.00010/-)		
*H*	B	349	*AP1S3* (c.81G>C/0.00020/0.00044)^∧^, *POLE* (c.3378+10A>G/0.00340/0.00462)^∧^	*CLPB* (c.1831G>A/0.00150/0.00176), *IGLL1* (c.512A>G/0.01980/0.01166), *IL17RC* (c.257_268delGGGGCAAGAGCT/0.01020/UnK), *MRE11* (c.1724G>A/0.00070/0.00066), *SLC35C1* (c.526A>G/UnK/-), *TTC37* (c.1124C>T/0.00020/0.0011), *UNC13D* (c.1637A>T/UnK/-)		
*I*	B	349	*CFTR* (c.1210-7_1210-6insA/UnK/UnK), *MEFV* (c.1105C>T/0.07160/0.05873, c.442G>C/0.31500/0.25781)	*CCBE1* (c.602C>T/0.00210/0.00132), *ERCC6L2* (c.3449G>A/0.01790/UnK), *FAT4* (c.11498A>G/0.00030/UnK), *HPS6* (c.632G>C/0.00600/0.0088), *IGLL1* (c.197G>A/0.00370/0.00242), *IL17RC* (c.1067C>T/0.00060/0.0011), *LRBA* (c.3064G>A/0.00100/0.0022), *NSMCE3* (c.325G>A/UnK/-), *STXBP2* (c.953C>T/0.00500/0.00616)		
*J*	U	349		*PRKDC* (c.7130-5G>A/0.00010/0.00044, c.722-6C>T/UnK/-)^#^, ^∧^, *CARD9* (c.661G>A/0.00340/0.00154), *EPG5* (c.3391A>G/0.01220/0.01276), *FOXN1* (c.331A>C/UnK/-), *IGLL1* (c.334G>A/0.00240/0.00198), *IL17RC* (c.1889C>A/UnK/-), *LYST* (c.8368A>C/0.01050/0.0143), *MOGS* (c.70C>T/0.00750/-)		
*K*	B	349	*TNFRSF13B* (c.17G>A/0.00020/0.00044)^∧^, *CARD11* (c.1017+10G>A/0.00130/0.00088) (splicing)	*CR2* (c.641G>A/0.00460/0.00176), *DOCK2* (c.4346G>A/0.00070/0.00154), *FOXN1* (c.1349G>A/UnK/-), *MASP1* (c.64G>A/0.01250/0.01122)		*POLE* (c.504A>T/0.00001/UnK)
*L*	U	423	*SAMD9L* (c.1886G>A/0.0001997/-), *CD46* (c.901+5A>G/0.000004/-), *ORAI1* (c.12G>T/0.0117188/-)	*NBAS* (c.1352A>G/0.0000548/-), *ERCC6L2* (c.1969A>G/0.0044987/0.00286), *IL10RB* (c.131C>T/0.0076923/0.00352), *LRBA* (c.5149G>A/0.0012512/0.00088), *BRCA1* (c.3974G>A/UnK/0.00022), *CD19* (c.1609C>A/0.000641/0.00022), *MASP1* (c.1888A>G/0.0001997/-), *RAB27A* (c.560G>A/0.0070675/0.00132), *SLCO1B1* (c.452A>G/0.0046/0.0044) (DR)^@^		
*M*	B	423	*MEFV* (c.442G>C/0.315/0.25781)^∧^	*AP3D1* (c.2938-8A>C/0.0004/-), *DCLRE1C* (c.850A>G/0.0004351/0.00088), *SPINK5* (c.1540C>A/0.0019256/0.00022)	*IL2RG* (c.1058A>C/UnK/-) (hemi)^$^	
*N*	B	423	*CLCN7* (c.352G>A/0.000641/-)^∧^	*FANCD2* (c.1278+3_1278+6delAAGT/0.000004/-) (splicing), *PMS2* (c.1276C>T/UnK/-), *ZNF341* (c.143-6C>T/0.0089744/0.00462) (splicing), *RELB* (c.1567G>A/0.0027/0.00616), *HPS4* (c.1947G>A/0.0176164/0.02266)		*TLR4* (c.910T>C/0.0001/-)
*O*	U	423	*MEFV* (c.442G>C/0.315/0.25781)^∧^, *IRF8* (c.1157G>T/UnK/-)^∧^	*RAG1* (c.650C>A/0.0006427/-), *CSF2RB* (c.2222C>T/0.000008/-), *SLX4* (c.1258G>C/UnK/-, c.5072A>G/0.0025674/0.00132), *IL2RB* (c.1109C>T/0.0057766/0.00198), *IL17RC* (c.260_271delGCAAGAGCTGGG/0.012837/0.0055), *IL12RB1* (c.1384G>T/0.0032134/0.00682), *LIG1* (c.746G>A/0.0198973/0.00528), *HPS6* (c.277G>T/0.0040791/0.00132)		
*P*	U	423	*RTEL1* (c.1286C>G/UnK/-)^@@^, *KMT2A* (c.2162G>C/UnK/-)∧, *MEFV* (c.442G>C/0.315/0.25781)∧	*FANCC* (c.55C>G/UnK/-), *IL2RB* (c.1192G>A/0.0045/0.00462), *IGLL1* (c.197G>A/0.0037/0.00242), *NT5E* (c.141C>G/0.0021/0.00198), *LYST* (c.452A>G/0.0025674/0.00154)		

& Freq-normal: frequencies of the gene variant in the normal population, the highest frequency was taken among 1,000 genomes, ESP6500 (NHLBI Exome Sequencing Project), EXAC (The Exome Aggregation Consortium), and EXAC-EAS (EXAC of 4,000 eastern Asian descent).

&& Freq-Chinese: frequencies of the gene variant in the in-house Chinese reference population data of MyGenostics Inc. (Beijing, China) that was archived from 2,273 normal individuals.

* Uncertain inheritance.

∧ Mutations were confirmed using Sanger sequencing.

$ Mutations were confirmed using Sanger sequencing in both the patient and her daughter.

@@ Mutations were confirmed using Sanger sequencing in both the patient and her father.

# Could not be predicted due to the excessively long gene sequence.

@ DR, double autosomal recessive inheritance.

AD, autosomal dominant inheritance; AR, autosomal recessive inheritance; B, bilateral MD; U, unilateral MD; Ver, version of gene sequencing with various candidate genes; XLR, X-linked recessive inheritance; (-), no mutation was detected.

## 4. Discussion

In the present preliminary study, we detected variants in multiple genes involved in the immune response in a small series of Chinese patients with sporadic definite MD. The results indicate that multiple genetic variants exist in most patients with sporadic MD, and the related gene products are involved in the regulation of innate and adaptive immunity.

There is a broad disease spectrum associated with the detected definite pathological variants contributing to clinical phenotypes in human diseases and definite pathological variants with respect to the structure and function of translated proteins. *MEFV* encodes the protein pyrin, which has been shown to act as a regulator of inflammation mediated by IL-1β. A recent study showed that treatment of cultured human endolymphatic sac epithelial cells with IL-1β reduced epithelial sodium channel expression and the associated current indicating inflammation may disrupt fluid absorption through the endolymphatic sac (25). Mutations in *MEFV* have been defined as variants specific to autoinflammatory diseases and have also been detected in autoimmune diseases (26, 27). Mutations in *COL7A1*, which encodes type VII collagen, have been reported to cause dystrophic epidermolysis bullosa, an inherited epidermolysis bullosa that demonstrates activated autoimmunity and inflammatory responses (28). Mutations in *COL7A1* were reported to potentially overlap with the *TMIE* gene that is involved in the mechanoelectrical transduction of cochlear hair cells and detected in a case of bilateral auditory neuropathy (29). It is possible that gene products of *TMIE* were disrupted by the abnormal stop codon. However, the phenotype of patient M did not fall in the category of auditory neuropathy spectrum disorder that might become atypical at an advanced stage. It is possible that the current patient had a comorbidity of MD and an auditory neuropathy spectrum disorder. *ADA* is a key enzyme of the purine salvage pathways, and absent or impaired *ADA* function leads to the accumulation of 2′deoxyadenosine and deoxyadenosine triphosphate, which are toxic metabolites of adenosine, in rapidly proliferating cells such as lymphocytes. This accumulation results in severe combined immunodeficiency. *ADA* deficiency has also been reported to be associated with sensorineural hearing loss (30). ADA^−/−^ mice developed hearing deficits resulting from extensive damage to cochlear hair cells and impairment of immune cells; these effects were improved by enzyme replacement therapy (31). *COLEC11* encodes the CL-K1 component of collectins belonging to a family of pattern recognition molecules with key roles in host defense, tissue homeostasis, and embryogenesis. Mutations in *COLEC11* may cause 3M syndrome, which is associated with craniofacial dysmorphism, intellectual disability, and genital, renal, and limb abnormalities (32). *RAG2* encodes lymphoid-specific recombinase that initiates V(D)J recombination, a site-specific chromosomal rearrangement process responsible for the immense diversity of TCRs and BCRs, and mutations in *RAG2* may cause autoimmune diseases (33). *BLM* encodes BLM helicase or RECQL3, which is a nucleolar protein essential for the integrity and stability of DNA. Mutations in *BLM* result in Bloom's syndrome, an autosomal recessive disease with abnormal immunoreaction (34). *RNF31* is a linear ubiquitin chain assembly complex component and regulates cell survival by inducing linear ubiquitination of NF-κB signaling components. Mutations in *RNF31* may cause immunodeficiency, autoinflammation, amylopectinosis, and lymphangiectasia (35). *FAT4* encodes a large protein with extracellular cadherin repeats, EGF-like domains, and laminin G-like domains that play a role in tumor suppression and planar cell polarity. Mutations in *FAT4* may cause neuronal defects in addition to the well-known van Maldergem syndrome, characterized by intellectual disability, periventricular heterotopia, characteristic facial features, camptodactyly and syndactyly, small kidneys, osteoporosis, and tracheal anomalies (36). The *PEPD* gene encodes prolidase, a protease enzyme involved in metal-associated catalytic mechanisms. Mutations in *PEPD* may cause symptoms such as ulceration and other dermatologic manifestations, telangiectasis, impetigo-like eruptions, lesions, necrotic papules, intellectual disability, respiratory tract infections, and facial dysmorphisms (37). *TNFRSF13B* encodes the transmembrane activator, calcium-modulator, and cyclophilin ligand interactor involved in class-switch recombination and the maintenance of memory and plasma B cells. Mutations in *TNFRSF13B* have been identified in common variable immunodeficiency (38). *VPS13B* encodes the VPS13B protein, which is important for Golgi structure maintenance, and genetic variants have been linked to the autosomal recessive neurodevelopmental disorder Cohen syndrome, which is characterized by intellectual disability, developmental delay, microcephaly, a characteristic facial appearance, progressive retinopathy, myopia, and/or neutropenia (39).

The detected limited and likely pathological variants seemed relevant. *TLR3* plays a key role in immune-mediated lupus nephritis by inducing sustained type I interferon activation (40). *RAB27A* controls the exosome secretion pathway and the terminal transport of lytic granules to immune synapses (41).

Regarding the limited pathological variants detected in five out of the 16 patients, *EXTL3* is a member of the exostosin family of glycosyltransferases that regulate glycosylation, a process by which glycans are attached to both proteins and lipids in the ER or Golgi complex. It has been reported that biallelic missense mutations in *EXTL3* cause neuroimmunoskeletal dysplasia syndrome, with some individuals presenting with a lack of CD4^+^ and CD8^+^ T cells (42). *MTHFD1* encodes a trifunctional protein essential for folate metabolism, and the defects are associated with severe combined immunodeficiency (43). *FANC* encodes FANC proteins that participate in repairing DNA interstrand crosslinks and are important for immunity and organellar homeostasis (44). Variants in the *FANC* gene cause Fanconi anemia, characterized by congenital abnormalities, bone marrow failure, and cancer predisposition (45). *LPIN2* encodes the protein lipin 2, a magnesium-dependent phosphatide phosphatase enzyme that associates with membrane lipids and localizes to organelles. Recessive loss-of-function mutations in *LPIN2* are involved in the dysregulation of innate immune responses resulting in systemic inflammation and osteomyelitis, including Majeed syndrome (45). *NBAS* gene encodes the NBAS protein, which functions as a component of an ER tethering complex involved in retrograde Golgi–ER transport. Variants in *NBAS* cause a disruption in Golgi-ER transport and formation of large transport vesicles at the ER exit site, induced ER stress and inflammation (46). *FCN3* gene encodes the Ficolin-3 protein, which is expressed in the lung and liver and is a recognition molecule in the lectin pathway of the complement system. Variants in *FCN3* may cause a deficiency in ficolin-3-dependent complement activation and lead to recurrent infections (47).

Mutations in the abovementioned genes are involved in various diseases, impairing the immune response at various stages, from the level of regulating integrity and stability of DNA, posttranslational modification, and trafficking of proteins and exosome secretion pathway to the final step of the immune response. Although the variants detected in patients with MD in the present study are not identical to those detected in other diseases, these variants are harmful to the gene products and may cause pathological changes in the individual according to current database data. Regarding *RNF31* variants involved in linear ubiquitination of NF-κB signaling components, regulatory variants in the NFKB1 gene have been reported to modify hearing outcomes in patients with MD and unilateral sensorineural hearing loss (48). The TLR3 signaling pathway has also been implicated in the innate immune response in the human endolymphatic sac (49). Patient A was also diagnosed with hyperthyroidism and secondary hypothyroidism with negative antibodies against thyroglobulin protein, thyroid peroxidase, and thyroxine receptor, indicating an autoinflammatory condition (11). Patient N has the comorbidity of ankylosing spondylitis, a chronic inflammatory arthritis that may cause inner ear issues. Patient P has the comorbidity of ulcerative colitis, an autoimmune disease that may also be accompanied by MD. We do not expect to discover genetic variants specific to MD because both autoimmunity and autoinflammation may involve in multiple organs. However, individuals carrying the abovementioned genetic variants may be susceptible to developing MD with the appearance of other triggers.

A missense variant in *PRKCB* was reported to segregate low-frequency sensorineural hearing loss in an autosomal dominant family with MD. *PRKCB* encodes a serine and threonine-specific protein kinase involved in many different cellular functions, such as neutrophil chemotaxis, melanoma cell growth and proliferation, or induction of apoptosis in endothelial cells (50). Our recent study demonstrated that G-CSF, IL8, and HGF are involved in the development of neutrophil extracellular traps and, through various mechanisms, influence the functions of macrophages, lymphocytes, and dendritic cells, which among others, are key players in the development of EH and MD (51). The behavior patterns of patients with MD showed strong tendencies toward engrossment, self-inhibition, feeling pressed for time, and aggressiveness compared to those of controls that engender day-to-day stress and frustration (52). A recent study on the surgical results of endolymphatic sac drainage surgery and changes in stress-induced plasma arginine-vasopressin levels showed that a gradual plasma vasopressin level elevation in recurrent MD after surgery suggested a link between symptom attacks and internal stress (53). Although endolymphatic sac drainage surgery was reported to be non-specific for MD and the outcome of the surgery might be linked to other biological effects such as shear stress induced by drilling the mastoid (54, 55), the results demonstrating patients without any symptom attacks having significantly low levels of plasma arginine-vasopressin compared to those with symptoms suggest a strong link between symptom attacks and stress (53). It was also reported that acute stress affects endocrine, immune, and metabolic functions in humans, and mood plays a causal role in the modulation of responses to acute stress (56). Individuals carrying the abovementioned genetic variations may be susceptible to developing inflammation and EH under stress conditions.

In addition to variants in immune genes, those in other genes, such as *DTNA, FAM136A, SEMA3D*, and *DPT*, relevant to the formation or maintenance of inner ear structures and inner ear ionic homeostasis have been detected in familial MD. Those variations demonstrate genetic heterogeneity in MD (57).

There are obvious limitations in the current study. We were unable to compare our results to a previous report on genetic variants detected in 890 MD patients because different target genes were selected. In the previous report, most of the 69 genes were selected from a list for monogenic sensorineural hearing loss although additional genes were added according to previous results on familial MD among others (58). In the present study, 269–423 target genes were selected by focusing on autoimmunity and autoinflammation. The Chinese individuals may also carry different genetic variants compared with the Spanish group. The small sample size makes it impossible to segregate the phenotype or to perform a burden test to search for the association. A future study using the current targets combined with targets related to the inner ear function based on a large sample selection is needed.

## 5. Conclusion

In conclusion, the current preliminary study results suggest that patients with sporadic MD carry multiple genetic variants involved in immune regulation, which might render these patients susceptible to developing inflammation via autoimmune and autoinflammation mechanisms and EH upon stress. Therefore, multiple hits might be involved in triggering the occurrence of MD. However, a large sample size is needed to replicate these findings.

## Data availability statement

According to national legislation/guidelines, specifically the Administrative Regulations of the People's Republic of China on Human Genetic Resources (http://www.gov.cn/zhengce/content/2019-06/10/content_5398829.htm; http://english.www.gov.cn/policies/latest_releases/2019/06/10/content_281476708945462.htm), no additional raw data is available at this time. Data of this project can be accessed after an approval application to the China National Genebank (CNGB, https://db.cngb.org/cnsa/). Please refer to: https://db.cngb.org/, or email: CNGBdb@cngb.org for detailed application guidance. The accession code CNP0004038 should be included in the application.

## Ethics statement

The studies involving human participants were reviewed and approved by the Ethical Committee of Shanghai Changhai Hospital (CHEC2020-107). The patients/participants provided their written informed consent to participate in this study.

## Author contributions

JZ made substantial contributions to the conception and design of the work, acquisition, analysis and interpretation of data, and drafting the work. GZ, HL, and ZZ participated in clinical work and acquisition of data. QZ performed the audiological measurements and participated in acquisition of data. IP and AM participated in drafting the work. All authors contributed to the article and approved the submitted version.

## Glossary

3M syndrome: Carnevale, Mingarelli, Malpuech, and Michels syndrome
ADA: adenosine deaminase
BCR: B-cell receptor
BLM: Bloom syndrome
COLEC11: collectin subfamily member 11
COL7A1: collagen type VII α1 chain
DTNA: dystrobrevin α
DPT: dermatopontin
EH: endolymphatic hydrops
ERCC6L2: ERCC excision repair 6 Like 2
ER: endoplasmic reticulum
ESCS: high-dose steroid delivery to the surface of the intact endolymphatic sac and incus
EXTL3: exostosin like glycosyltransferase 3
FAM136A: family with sequence similarity 136 member A
FANCA: Fanconi anemia, complementation group A
FAT4: FAT atypical cadherin 4
FCN3: Ficolin 3
G-CSF: granulocyte colony-stimulating factor
Gd-DTPA: gadolinium-diethylenetriamine pentaacetic acid
HGF: hepatocyte growth factor
hT_2_W-FLAIR: heavily T_2_-weighted 3-dimensional fluid-attenuated inversion recovery
hT_2_W-FLAIR-ZFI: hT_2_W-FLAIR reconstructed with magnitude and zero-filled interpolation
IL-1β: interleukin-1β
IL-1R: interleukin-1 receptor
IL2RG: interleukin-2 receptor-γ
IL6 and 8: interleukin-6 and 8
ITDex: intratympanic dexamethasone
LPIN2: lipin 2
MD: Meniere's disease
MEFV: Mediterranean fever innate immunity regulator
MIIRMR: hT_2_W-FLAIR with medium inversion time inversion recovery imaging and magnitude reconstruction
MOGS: mannosyl-oligosaccharide glucosidase
MRI: magnetic resonance imaging
MTHFD1: methylenetetrahydrofolate dehydrogenase, cyclohydrolase, and formyltetrahydrofolate synthetase 1
NBAS: neuroblastoma amplified sequence
PEPD: Peptidase D
POLE: DNA polymerase ε
PRF1: perforin 1
RAG2: recombination activating 2
RNF31: ring finger protein 31
SEMA3D: semaphorin 3D
SPACE: T_2_-sampling perfection with application-optimized contrasts using a flip angle evolution
TCR: T-cell receptor
TIRAP: Toll-interleukin 1 receptor domain containing adaptor protein
TLR3 and TLR4: toll-like receptor 3 and 4
TNF-α: tumor necrosis factor-α
TNFR1: tumor necrosis factor receptor 1
TNFRSF13B: tumor necrosis factor receptor superfamily member 13B
TNFSF12: tumor necrosis factor ligand superfamily member 12
UNC13D: unc-13 homolog D
VPS13B: vacuolar protein sorting 13 homolog B.
